# Surveying the attitudes of transsexual patients referring to Tehran Institute of Psychiatry toward doctors’ empathy, Iran, 2011-2012

**Published:** 2015-11-07

**Authors:** Ghazaleh Ahmadi Jazi, Mehrdad Eftekhar, Pezhman Mobasher, Saeedeh Saeedi Tehrani, Khosro Ahmadi, Maria Rastgouy Fahim

**Affiliations:** 1 Researcher, Medical Ethics and History of Medicine Research Center, Tehran University of Medical Sciences, Tehran, Iran;; 2 Associate Professor, Mental Health Research Center, Iran University of Medical Sciences, Tehran, Iran;; 3 Skin and Stem Cell Research Center, Department of Dermatology, Tehran University of Medical Sciences, Tehran, Iran;; 4 Medical Ethics PhD Candidate, Department of Medical Ethics, Faculty of Medicine, Tehran University of Medical Sciences, Tehran, Iran;; 5 Department of Pediatrics, Mashhad University of Medical Sciences, Mashhad, Iran;; 6 Department of Obstetrics and Gynecology, Shahid Beheshti University of Medical Sciences, Tehran, Iran.

**Keywords:** *patients’ attitude*, *sexual identity disorder*, *transsexual*, *empathy*

## Abstract

Physicians’ knowledge of therapy and counseling stands among the most important issues in the viewpoints of clients who refer to psychiatric centers. Transsexual patients are very important in this regard. The goal of this research is to study their attitude toward doctors’ empathy.

A group of transsexual patients who referred to the Tehran Institute of Psychiatry, Iran, answered the Jefferson Scale of Empathy. The relationship of the patients’ age, gender, education level, and lifestyle with their attitude was measured.

This study was conducted on 40 patients, including 16 women (40%) and 24 men (60%). In terms of education, 8 patients had a degree below high school diploma (20%), 9 had high school diploma (22.5%), and 23 patients were university students or of higher education level (57.5%). Among these patients, 6 were unemployed (15%), 10 were students (25%), and the rest were employed. Moreover, 8 participants lived alone (20%), 5 lived with their friends (12.5%), and 27 lived with their family (67.5%). Gender had no influence on the average score of the questionnaires, yet level of education had some influence. Lifestyle also had a significant influence on the patients’ attitude. On the other hand, patients whose problems began before the age of 12 had lower score than others.

Experienced psychologists in referential centers can express greater levels of empathy to specific diseases and this trend is very effective on the patients’ cooperation level. In order to create an effective relationship between physicians and patients, the efficiency of the health system and increasing satisfaction of specific patients should be considered.

## Introduction

A good relationship between physicians and patients is the basis of a good medical care (1-3). Researches indicate that most medical diagnoses and treatment decisions are based on information obtained from interviews and a good physician-patient relationship is the basis of medical interviews. An effective relationship between physicians and patients is a skill and physicians and patients should have an empathetic behavior to realize it (4). Therefore, establishment of an empathetic relationship between physicians and patients is a key to success in a treatment process. More complaints against physicians have been related to the manner of their relationships with patients than their ability and skill (5). Establishment of effective relationships not only increases patient satisfaction, but also has an important role in the enhancement of the result of medical action (6).

Empathy means sensitivity to the mental condition of other individuals and the ability to understand and know it. Today, professional empathy is emphasized as a means of understanding the mental and emotional status of patients and its effect on success in clinical activities (7).

Empathy also greatly helps in increasing patients’ satisfaction and compliance, and therefore, facilitating treatment method by raising the patients’ confidence in physicians (8).

Therefore, empathy is required and necessary for a good relationship between physicians and patients, maintaining confidence in physicians, and having a professional behavior. In addition, having empathy is stated as a professional principle and obligations. This issue and its evaluation will greatly help in achieving health goals. Evaluation of patients in terms of such empathy will also help in appraisal of health services.

Unfortunately, many physicians refuse to conduct such evaluations with the justification of not having enough time to establish a good relationship, and thus, to empathize with patients. This is observed more in specific diseases. Previous researches have indicated the existence of serious problems in this regard in some countries (9) and that this weakness (physicians’ lack of empathy) has harmful effects on the physical, mental, economic, and social dimensions of health care (10).

Based on the Diagnostic and Statistical Manual of Mental Disorders, 4^th^ Edition (DSM-IV) measures, annually 1 per 30,000 adult males and 1 per 100,000 adult females seek sex-reassignment surgery. Based on statistics provided by clinics in Amsterdam, this surgery is used for 1 per 10,000 males and 1 per 30,000 females. However, van Kesteren et al. consider these figures to be higher (11).

Empathy is the easiest way of effectively taking care of patients, and understanding their emotional and verbal behaviors with an attempt to comprehend their emotions, desires, and words. The main key to improving the curative effect of the patient-doctor relationship is empathy. It has been proven that improving communications with patients based on empathy and correct comprehension can bring about higher quality care and treatment for patients. Although the importance of empathy might be quite clear, its definition and influence on the patient-doctor relationship still needs to be explained. 

The first person who believed in the importance of empathy in the patient-doctor relationship was Carney Rogers. He proved the importance of empathy as a factor that promotes the curative influence. According to Rogers empathy is the correct comprehension of another individual’s inner experience (12). 

Truax also defined empathy as the correct understanding of the current client’s feelings and taking a structured path to having dialogue with patients (13). Based on this definition, many people believe that empathy is a skill and an attitude, and the ability to comprehend the emotions of other people and the cause of such emotions (14). Random information has indicated a relationship between empathy and routine behavior. The same information has also proven the beneficial effects of empathy on patients’ health, better cooperation of patients, reduction of legal problems concerning doctors, and higher levels of doctor and patient satisfaction (15). 

The relationship between empathy and empathetic and assisting behavior is defined in the 4 categories of sentimentality, morality, recognition, and behavior (16). 

Sentimentality is the ability to experience the emotional state of another person. Morality is necessary for the sense of humanity and practice of empathy. Recognition is the ability to correctly understand and comprehend the attitudes of another person. Behavior is the understanding the similarities in behaviors of the opposite individual.

Patterson has also described empathy in 4 steps the assisting individual’s talent to receive signals from another individual, to put oneself in place of another individual, mutual understanding, and patients’ understanding of the assistant’s perception. Based on this definition, empathy falls into 4 categories of emotional, receptive, communicative, and relational (17). According to the Internal Medicine community, empathy means understanding the emotional state of an individual without really being in that situation. This definition shows that empathy is a mixture of the 2 components of intelligence and emotion. Occupational empathy is a form of empathy which is mostly based upon intelligence and recognition aspects rather than emotional aspects. 

In a study, doctors were asked to define empathy. They defined it as: “putting myself in the patient’s shoes.” In that study, women emphasized the emotional aspect of empathy, while men focused their attention on the importance of empathetic behaviors (like accompanying the patient, the possibility of direct phone calls, prescribing cheaper medicines, and etc.) (18, 19).

To our knowledge, no study exists that shows the prevalence of transexuality in the Iranian population. Yet, we cannot hide the problems these individuals have in various stages of their life. The patients usually treat their condition as a secret and hide it even from their close circle of friends. Many of these patients do not even go to psychiatric and psychologist centers for treatment. Among patients who come to psychiatric institutes for treatment and consultation, one of the most important issues is probably the level of understanding of responsible patients about issues of therapy and consultation. Thus, we decided to study the empathy level of doctors from the view of transsexual patients in a psychiatric institute in Iran. Thus, the main goal of this study was to survey the attitude of patients referred to the Tehran Institute of Psychiatry, Iran, in 2011 toward doctors’ empathy. Variables of age, education, unemployment, gender, sex reassignment, the age at which sex-associated problems began, and the rejection level from family were considered. Therefore, evaluation of patients’ understanding of empathetic behavior and studying the rate of understanding and empathy between physicians and patients, especially patients with special conditions like transsexuals, can increase communication skills and change individuals’ attitudes. It can also be a type criterion for evaluation of physicians’ professional obligations and enhancement of health services in this area. 

## Materials and methods

The present research was a cross-sectional study.

**Statistical sample and population**

The statistical population of this study consisted of transsexual patients who referred to the Tehran Institute of Psychiatry. Tehran Institute of Psychiatry is one of the institutions to which forensic medicine organizations introduce some transsexual patients for more evaluation. All patients referring to the institute during 2011-2012 were included in the study using non-random simple sampling (based on convenient samples). A sample of 40-50 patients was selected. 

**Data collection tools and methods**

The data collection tool was a questionnaire designed by the researchers. It included questions about patients’ demographic information, such as age, employment status, lifestyle, education, and the age at which sexual problems started, and 5 questions concerning patients’ attitude toward doctors’ empathy (20). These 5 questions were adopted from the Jefferson Scale of Empathy which was modified by Hojat et al. (21). In the study by Hojat et al., some changes were made to the Jefferson Scale of Empathy so that it could be used by physicians and other therapists. Thus, this modified version has been used for measuring the perception of patients about physician empathy level. Reliability and validity of the questionnaire were confirmed by Hojat et al. (21). Moreover, in this study, Cronbach’s alpha was used to ensure the reliability of the questionnaire. The Cronbach's alpha of the research tool was 0.85 which confirmed its reliability. Finally, the validity of the questionnaire was confirmed using content validity. 

**Data Analysis**

The data were transferred to SPSS software (version 19, SPSS Inc., Chicago, IL, USA) and analyzed. The average age of the patients was calculated. The average questionnaire score for patients in terms of variables of age, sex, occupation, and education level was calculated. Chi-square statistical test was used to compare qualitative variables and analysis of variance (ANOVA) was used to compare quantitative variables. The confidence level of the study was 95% and all P values of less than 0.05 were considered to be significant. 

## Results

**Figure 1:**
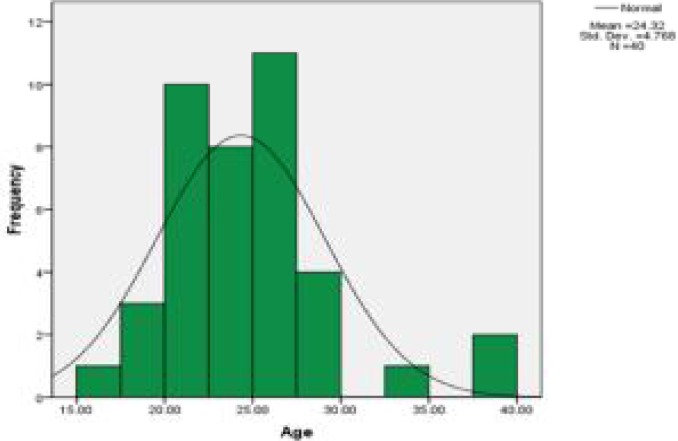
Age distribution of patients

The present study was conducted on 40 patients, including 16 women (40%) and 24 men (60%) with the average of 24.32 ± 4.77. The average age of men in this study was 24.50 ± 5.6 years and the average age of women was 24 ± 3 years which did not differ significantly. On the other hand, the majority of patients were younger than 30. There were only 3 patients above the age of 30. Figure 1 shows the age distribution of patients in this study. 

In terms of education, 8 patients had a degree below high school diploma (20%), 9 had high school diploma (22.5%), and 23 were students or had degrees higher than high school diploma (57.5%). In addition, 6 patients were unemployed (15%), 10 were students (25%), and others had occupations such as teacher and barber. Based on the answers to the questions, 8 people lived alone (20%), 5 lived with their friends (12.5%), and 27 lived with their families (67.5%). 

The average score of the Jefferson Scale of Empathy including 5 five-point questions (25 scores in total) was 19 ± 2.13 for women and 19 ± 2.6 for men. Hence there was no significant difference between the scores of the two genders. Table 1 shows the frequency of the scores of the two genders (P < 0.94).

**Table 1:** Frequency of the scores of the two genders

Gender	Average	Range	*P*-value
Female	19 ± 2.13	15-22.5	0.94
Male	19 ± 2.6	13-25	
Total	19 ± 2.38	13-25	

 The average score of patients with a degree below high school diploma, high school diploma, and degree above high school diploma was 21.12 ± 1.95, 18.4 ± 2.46, and 18.5 ± 0.2.17, respectively. The difference was significant. Table 2 shows the average frequency of scores for each level of education in patients (P < 0.02).

**Table 2:** The average frequency of scores for each level of education

Level of education	Average	Range	*P*-value
Below high school diploma	21.12 ± 1.95	9-25	0.02
High school diploma	18.4 ± 2.46	15-22.5	
Above high school diploma	18.5 ± 2.17	13-22.5	

Table 3 shows the frequency distribution of the average scores of the Jefferson Scale of Empathy for each occupation. Based on the occupation status, the average score for unemployed patients, students, and for employed participants was 17.8 ± 0.1.85, 18 ± 2.55, and 19.8 ± 2.3, respectively. The difference was significant (*P* < 0.03).

**Table 3:** The frequency distribution of the Jefferson Scale of Empathy score for unemployed participants, students, and employed participants

Occupational status	Average	Range	*P*-value
Unemployed	18 ± 2.55	13-20	0.03
Student	17.8 ± 1.85	15-20.5	
Employed	19.8 ± 2.3	16-22	

In terms of lifestyle, those who lived alone, those who lived with their friends, and those who lived with their family obtained the scores 18.7 ± 0.2.6, 20.6 ± 0.2.77, and 18.8 ± 0.2.23, respectively. The differences observed among scores in terms of lifestyle were not significant. 

Among the 40 transsexual patients studied, 25 patients (62.5%) said their problems started before the age of 12, and 15 patients (37.5%) said their problems started after the age of 12. The average score of Jefferson Scale of Empathy for patients whose problems started before the age of 12 and after the age of 12 was 18.4 ± 0.2 and 20 ± 0.2.6, respectively. The difference observed here was significant (*P* < 0.038). 

***Discussion and conclusion***

The goal of the present study was to evaluate the empathy level of transsexual patients who referred to an Iranian psychological center. The Jefferson Scale of Empathy was used to study the empathy level of patients. Based on the information gained from this scale, the majority of patients who referred to the Tehran Institute of Psychiatry were less than 30 years of age. The majority of them were students and only a small percentage of them were unemployed. This shows that the population studied was mostly educated and independent. The results revealed that the majority of participants had education levels above high school diploma. 

The patients were asked to complete two sample forms, one for the institute’s psychiatrists and one for other physicians. This was used to evaluate the attitude of patients before and after attending institution. In previous studies, psychiatry residents showed high empathy levels compared to other residents (22). In this study, the patients gave an average score of 17.6 ± 3.6 to other institutions and 20.5 ± 2.6 to the Tehran Institute of Psychiatry.

In this study, 62.5% of the patients faced problems before 12 years of age, before puberty. This finding was in line with our hypotheses. The others faced problems after puberty. This showed the early and late symptoms of patients. Thus, according to our expectation, the patients’ symptoms start earlier than 12 years of age and the status of other patients is doubtful. The patients with late symptoms need a different type of empathy.

The important finding of this study was that the patients observed a higher level of empathy in the institute’s psychiatrists than their previous psychiatrists. As the subject conducting interviews in this study was not one of the therapists of the participants, reporting higher levels of empathy by the patients is unlikely to affect therapists’ satisfaction or their concern of therapy.

 This finding can be explained in terms of these psychiatrists’ higher experience of this disorder, as a result of which they can show their empathy better. Thus, training health service providers on transsexualism is of great importance.

The study results showed that there is no difference among genders regarding attitude toward empathy. This finding is consistent with the results of the study by Owen-Anderson et al. (22). Their results showed that there is no difference regarding empathy between healthy girls and transsexual boys (22).

The results of the investigation of empathy based on education showed that patients with low education level reported high empathy. This was due to the effortless communication between the physician and patient and the greater confidence of the patients in their physicians. It can be said that transsexual patients with high education level should be further studied, as they have different needs from others based on their more sociable behavior and higher expectation level. These needs should be taken into consideration during interviews with these patients. Individuals with degrees below diploma have less expectation of empathy.

Moreover a difference was observed in the scores of the Jefferson Scale of Empathy based on the patients’ occupation. However, no specific reason was found for this finding in terms of occupational differences and empathy. The job and income of patients should be considered in the proposed solutions and we should behave differently with each transsexual patient depending upon their job, social expectation, and income. The results showed that there was no difference between the patients living alone and the patients living with their family or friends in terms of empathy. This is because of various factors in patients’ attitude toward physicians’ empathy. It was found that the patients living alone have higher mean age than others (28 years vs. 23 years) and their lifestyle had no impact on their attitudes.

The present research was appropriate in terms of the sample volume. Transsexual patients have hardly been noticed in the world and not studied in terms of empathy level. This is due to these patients’ lack of confidence in health centers or social obstacles. A major strength of this research was the selection of these patients. Another was the use of the Jefferson Scale of Empathy in this study. This study has utilized parts of this scale and 2 sample forms (for institute’s psychiatrists and other physicians) were completed. The present study also measured the relationship between empathy level and individual factors of transsexual patients. It has been proven that questioning transsexual patients about their expectations of and satisfaction level with the health system will lead to a closer relationship with the therapist and increase the treatment pace (phone calls with patients) (23).

Since having a good relationship leads to enhancement of the quality of health services and increase in satisfaction of patients, understanding the attitude of patients toward physicians’ empathy rate and its relevant factors can greatly help in obtaining their satisfaction. In addition, knowing influential factors and how they influence empathetic behavior, would help professionals to communicate with patients more effectively.

To name but a few restrictions of this research, we may refer to the use of questionnaires for data collection. Mingling these ideas and comprehensions with biases, thoughts, and prejudgments might influence the research results. It is recommended that future researches adopt a qualitative approach or a mixture of qualitative and quantitative approaches to identify the attitudes of patients toward doctors’ empathy. In future researches, it is recommended that a questionnaire be given to the doctors so that they can also measure the patients’ cooperation level and compliance with treatments. Furthermore, it is suggested that future studies be conducted with a greater sample volume based on other factors that influence empathy in the patient-doctor relationship. It also seems necessary to have an ethical guide for patients with mental disorders and for activists in this field to understand and know psychological issues in specific patients in order to have professional behavior in this regard. Moreover, teaching communication skills to professionals in this field can be helpful and seems necessary.
